# Supplementary material for: Global Proteomics to Study Silica Nanoparticle-Induced Cytotoxicity and Its Mechanisms in HepG2 Cells
Source: Biomolecules. 2021 Mar 2;11(3):375. doi: 10.3390/biom11030375 (PMC8000044; doi:10.3390/biom11030375)
Supplement: Supplementary file 1 [file biomolecules-11-00375-s001.pdf]

biomolecules

MDPI

*Electronic Supplementary Information (ESI) for*

# Global Proteomics to Study Silica Nanoparticle-Induced Cytotoxicity and Its Mechanisms in HepG2 Cells

**Sun Young Lee [1], In Young Kim [2], Min Beom Heo [2], Jeong Hee Moon [3], Jin Gyeong Son [1,*] and Tae Geol Lee [1,*]**

[1] Bioimaging Team, Safety Measurement Institute, Korea Research Institute of Standards and Science (KRISS), Daejeon 34113, Korea; tglee@kriss.re.kr
[2] Nano-safety Team, Safety Measurement Institute, Korea Research Institute of Standards and Science (KRISS), Daejeon 34113, Korea; mbheo@kriss.re.kr
[3] Disease Target Structure Research Center, Korea Research Institute of Bioscience and Biotechnology (KRIBB), Daejeon 34141, Korea; jhdal@kribb.re.kr
* Correspondence: yeskyoung@kriss.re.kr, Tel.: +82-42-868-5751 (J.G.S); tglee@kriss.re.kr; Tel.: +82-42-868-5003 (T.G.L)

**Table S1.** List of the exclusive proteins from HepG2 cells according to the exposure time of $SiO_2$ NPs by comparing the 0 h-group.

| #term ID | Term description | Count | FDR[1] |
|---|---|---|---|
| | **4h vs. 0 h (26)** | | |
| GO:0005740 | mitochondrial envelope | 13 | 8.28E-05 |
| GO:0005743 | mitochondrial inner membrane | 10 | 1.50E-04 |
| GO:0031966 | mitochondrial membrane | 12 | 1.50E-04 |
| GO:0098798 | mitochondrial protein complex | 8 | 1.50E-04 |
| GO:0098800 | inner mitochondrial membrane protein complex | 6 | 1.90E-04 |
| GO:0031967 | organelle envelope | 14 | 3.60E-04 |
| GO:0005747 | mitochondrial respiratory chain complex I | 4 | 5.00E-04 |
| GO:0043231 | intracellular membrane-bounded organelle | 48 | 6.40E-04 |
| GO:0005739 | mitochondrion | 15 | 1.10E-03 |
| GO:0000220 | vacuolar proton-transporting V-type ATPase, V0 domain | 2 | 3.60E-03 |
| GO:0005622 | intracellular | 56 | 3.60E-03 |
| GO:0032991 | protein-containing complex | 28 | 3.60E-03 |
| GO:0070013 | intracellular organelle lumen | 29 | 4.20E-03 |
| GO:0043226 | organelle | 51 | 5.20E-03 |
| GO:0043229 | intracellular organelle | 50 | 7.20E-03 |
| GO:0031090 | organelle membrane | 21 | 7.50E-03 |
| GO:0035577 | azurophil granule membrane | 3 | 7.70E-03 |
| GO:0032592 | integral component of mitochondrial membrane | 3 | 1.16E-02 |
| GO:0098796 | membrane protein complex | 10 | 1.17E-02 |
| GO:1902494 | catalytic complex | 11 | 1.64E-02 |
| GO:0005759 | mitochondrial matrix | 6 | 2.37E-02 |
| GO:0005763 | mitochondrial small ribosomal subunit | 2 | 2.92E-02 |
| GO:0005623 | cell | 58 | 3.38E-02 |
| GO:0005737 | cytoplasm | 45 | 4.02E-02 |
| GO:0031305 | integral component of mitochondrial inner membrane | 2 | 4.02E-02 |
| GO:0005654 | nucleoplasm | 19 | 4.35E-02 |
| | **10h vs. 0 h (40)** | | |
| GO:0005622 | intracellular | 133 | 9.84E-08 |

| | | | |
|---|---|---|---|
| GO:0005737 | cytoplasm | 117 | 9.84E-08 |
| GO:0043229 | intracellular organelle | 121 | 2.38E-07 |
| GO:0043231 | intracellular membrane-bounded organelle | 107 | 2.98E-06 |
| GO:1990904 | ribonucleoprotein complex | 22 | 3.63E-06 |
| GO:0043227 | membrane-bounded organelle | 112 | 4.11E-06 |
| GO:0098798 | mitochondrial protein complex | 11 | 1.60E-04 |
| GO:0019866 | organelle inner membrane | 15 | 2.90E-04 |
| GO:0005743 | mitochondrial inner membrane | 14 | 3.10E-04 |
| GO:0031966 | mitochondrial membrane | 17 | 4.00E-04 |
| GO:0005623 | cell | 135 | 5.40E-04 |
| GO:0005829 | cytosol | 59 | 5.90E-04 |
| GO:0044391 | ribosomal subunit | 8 | 6.00E-04 |
| GO:0005761 | mitochondrial ribosome | 6 | 1.30E-03 |
| GO:0032991 | protein-containing complex | 56 | 1.40E-03 |
| GO:0031967 | organelle envelope | 21 | 2.00E-03 |

[1]False discovery rate.

**Table S1.** (continued).

| #term ID | Term description | Count | FDR[1] |
|---|---|---|---|
| | **10h vs. 0 h** | | |
| GO:0070013 | intracellular organelle lumen | 58 | 2.50E-03 |
| GO:0005759 | mitochondrial matrix | 12 | 2.70E-03 |
| GO:0005739 | mitochondrion | 24 | 4.60E-03 |
| GO:0005819 | spindle | 9 | 9.90E-03 |
| GO:0031090 | organelle membrane | 40 | 1.00E-02 |
| GO:0035770 | ribonucleoprotein granule | 7 | 1.18E-02 |
| GO:0005654 | nucleoplasm | 40 | 1.75E-02 |
| GO:0005905 | clathrin-coated pit | 4 | 1.75E-02 |
| GO:0031981 | nuclear lumen | 45 | 1.75E-02 |
| GO:0005763 | mitochondrial small ribosomal subunit | 3 | 1.88E-02 |
| GO:0015935 | small ribosomal subunit | 4 | 1.88E-02 |
| GO:0005681 | spliceosomal complex | 6 | 3.07E-02 |
| GO:0098800 | inner mitochondrial membrane protein complex | 5 | 3.07E-02 |
| GO:1902494 | catalytic complex | 19 | 3.07E-02 |
| GO:0036464 | cytoplasmic ribonucleoprotein granule | 6 | 3.14E-02 |
| GO:0016604 | nuclear body | 13 | 3.20E-02 |
| GO:0000932 | P-body | 4 | 3.23E-02 |
| GO:0019774 | proteasome core complex, beta-subunit complex | 2 | 3.60E-02 |
| GO:0043232 | intracellular non-membrane-bounded organelle | 43 | 3.60E-02 |
| GO:0015934 | large ribosomal subunit | 4 | 3.76E-02 |
| GO:0000177 | cytoplasmic exosome (RNase complex) | 2 | 4.40E-02 |
| GO:0005747 | mitochondrial respiratory chain complex I | 3 | 4.48E-02 |
| GO:0030120 | vesicle coat | 3 | 4.48E-02 |
| GO:0010369 | chromocenter | 2 | 4.52E-02 |
| | **16h vs. 0 h (38)** | | |
| GO:0043231 | intracellular membrane-bounded organelle | 149 | 4.06E-11 |
| GO:0005622 | intracellular | 176 | 1.45E-10 |
| GO:0043227 | membrane-bounded organelle | 154 | 1.87E-10 |
| GO:0005737 | cytoplasm | 151 | 3.91E-09 |
| GO:0005829 | cytosol | 90 | 3.91E-09 |
| GO:0043229 | intracellular organelle | 158 | 5.44E-09 |

| | | | |
|---|---|---|---|
| GO:0043226 | organelle | 159 | 1.22E-08 |
| GO:0032991 | protein-containing complex | 85 | 3.88E-08 |
| GO:0031090 | organelle membrane | 63 | 2.29E-06 |
| GO:0070013 | intracellular organelle lumen | 83 | 6.68E-06 |
| GO:0005634 | nucleus | 97 | 1.30E-04 |
| GO:0031981 | nuclear lumen | 66 | 1.30E-04 |
| GO:0005623 | cell | 177 | 2.10E-04 |
| GO:0005654 | nucleoplasm | 57 | 5.10E-04 |
| GO:0031967 | organelle envelope | 27 | 5.40E-04 |
| GO:1990904 | ribonucleoprotein complex | 20 | 1.80E-03 |
| GO:0042175 | nuclear outer membrane-endoplasmic reticulum membrane network | 24 | 2.00E-03 |
| GO:0005730 | nucleolus | 22 | 2.50E-03 |
| GO:0005789 | endoplasmic reticulum membrane | 23 | 3.60E-03 |

[1]False discovery rate.

**Table 1.** (continued).

| | | | |
|---|---|---|---|
| GO:0098827 | endoplasmic reticulum subcompartment | 23 | 3.60E-03 |
| GO:0005840 | ribosome | 9 | 4.00E-03 |
| GO:0012505 | endomembrane system | 63 | 5.50E-03 |
| GO:0031205 | endoplasmic reticulum Sec complex | 2 | 1.04E-02 |
| GO:0005740 | mitochondrial envelope | 17 | 1.35E-02 |
| GO:0044391 | ribosomal subunit | 7 | 1.51E-02 |
| GO:0031301 | integral component of organelle membrane | 10 | 1.68E-02 |
| GO:0031966 | mitochondrial membrane | 16 | 1.68E-02 |
| GO:0043232 | intracellular non-membrane-bounded organelle | 57 | 1.68E-02 |
| GO:0098588 | bounding membrane of organelle | 33 | 1.68E-02 |
| GO:0098796 | membrane protein complex | 21 | 1.94E-02 |
| GO:0010008 | endosome membrane | 12 | 2.58E-02 |
| GO:0098805 | whole membrane | 27 | 2.95E-02 |
| GO:0005768 | endosome | 18 | 3.00E-02 |
| GO:0031965 | nuclear membrane | 9 | 3.75E-02 |
| GO:0005739 | mitochondrion | 26 | 4.47E-02 |
| GO:0031984 | organelle subcompartment | 27 | 4.53E-02 |
| GO:0032059 | bleb | 2 | 4.64E-02 |
| GO:0005783 | endoplasmic reticulum | 29 | 4.94E-02 |

[1]False discovery rate.

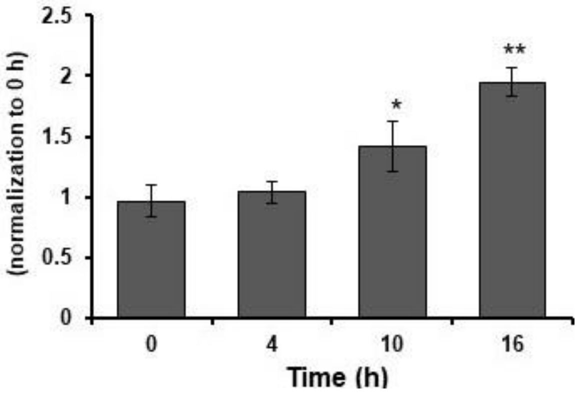


**Figure S1.** Determination of ROS levels in HepG2 cells over increase of exposure time (0, 4, 10, and 16 hours) to $SiO_2$ NPs. Data shown are mean ± standard deviation of three independent experiments. Asterisks indicate effects in comparison to 0 hour (* $p < 0.05$, ** $p < 0.005$).

**Table S2.** List of downregulated proteins associated with protein-protein interaction networks (PPI) in Figure 4.

| Gene name | Accession | Description | 4 h | 10 h | 16 h | PPI |
|---|---|---|---|---|---|---|
| MRPL11 | Q9Y3B7 | 39S ribosomal protein L11 | | | ○ | Ribosome |
| MRPL4 | Q9BYD3 | 39S ribosomal protein L4 | | | ○ | Ribosome |
| RPL10 | P27635 | 60S ribosomal protein L10 | | | ○ | Ribosome |
| RPL11 | Q9Y3B7 | 39S ribosomal protein L11 | | | ○ | Ribosome |
| RPL13 | P26373 | 60S ribosomal protein L13 | ○ | | | Ribosome |
| RPL17 | P18621 | 60S ribosomal protein L17 | ○ | | ○ | Ribosome |
| RPL18 | Q07020 | 60S ribosomal protein L18 | ○ | ○ | ○ | Ribosome |
| RPL18A | Q02543 | 60S ribosomal protein L18a | ○ | ○ | ○ | Ribosome |
| RPL19 | P84098 | 60S ribosomal protein L19 | | ○ | ○ | Ribosome |
| RPL22 | P35268 | 60S ribosomal protein L22 | ○ | ○ | ○ | Ribosome |
| RPL23 | P62829 | 60S ribosomal protein L23 | | | ○ | Ribosome |
| RPL3 | P39023 | 60S ribosomal protein L3 | ○ | ○ | ○ | Ribosome |
| RPL4 | P36578 | 60S ribosomal protein L4 | ○ | | | Ribosome |
| RPL7A | P62424 | 60S ribosomal protein L7a | ○ | | | Ribosome |
| RPL9 | P32969 | 60S ribosomal protein L9 | | ○ | ○ | Ribosome |
| RPS11 | P62280 | 40S ribosomal protein S11 | | ○ | ○ | Ribosome |
| RPS12 | P25398 | 40S ribosomal protein S12 | ○ | ○ | ○ | Ribosome |
| RPS13 | P62277 | 40S ribosomal protein S13 | ○ | ○ | ○ | Ribosome |
| RPS15 | P62841 | 40S ribosomal protein S15 | | ○ | | Ribosome |
| RPS17 | P08708 | 40S ribosomal protein S17 | ○ | ○ | ○ | Ribosome |
| RPS19 | P39019 | 40S ribosomal protein S19 | | | ○ | Ribosome |
| RPS2 | P15880 | 40S ribosomal protein S2 | ○ | | ○ | Ribosome |
| RPS20 | P60866 | 40S ribosomal protein S20 | | | ○ | Ribosome |
| RPS24 | P62847 | 40S ribosomal protein S24 | ○ | | ○ | Ribosome |
| RPS28 | P62857 | 40S ribosomal protein S28 | ○ | ○ | ○ | Ribosome |
| RPS3A | P61247 | 40S ribosomal protein S3a | ○ | ○ | ○ | Ribosome |
| RPS4X | P62701 | 40S ribosomal protein S4 X isoform | ○ | ○ | ○ | Ribosome |
| RPS7 | P62081 | 40S ribosomal protein S7 | ○ | | | Ribosome |
| RPSA | P08865 | 40S ribosomal protein SA | ○ | | | Ribosome |
| BCAS2 | O75934 | Pre-mRNA-splicing factor SPF27 | ○ | ○ | | mRNA splicing |
| BUD31 | P41223 | Protein BUD31 homolog | | | | mRNA splicing |
| CCAR1 | Q8IX12 | Cell division cycle and apoptosis regulator protein 1 | ○ | | ○ | mRNA splicing |
| CPSF3 | Q9UKF6 | Cleavage and polyadenylation specificity factor subunit 3 | ○ | | | mRNA splicing |
| CPSF4 | O95639 | Cleavage and polyadenylation specificity factor subunit 4 | ○ | | | mRNA splicing |
| CPSF7 | Q8N684 | Cleavage and polyadenylation specificity factor subunit 7 | | | ○ | mRNA splicing |
| CSTF1 | Q05048 | Cleavage stimulation factor subunit 1 | ○ | | | mRNA splicing |
| DDX42 | Q86XP3 | ATP-dependent RNA helicase DDX42 | ○ | | | mRNA splicing |
| DDX46 | Q7L014 | Probable ATP-dependent RNA helicase DDX46 | ○ | | | mRNA splicing |
| DHX9 | Q08211 | ATP-dependent RNA helicase A | ○ | ○ | | mRNA splicing |
| DNAJC8 | O75937 | DnaJ homolog subfamily C member 8 | | ○ | ○ | mRNA splicing |

**Table S2.** List of downregulated proteins associated with protein-protein interaction networks (PPI) in Figure 4 (continued).

| Gene name | Accession | Description | 4 h | 10 h | 16 h | PPI |
|---|---|---|---|---|---|---|
| EFTUD2 | Q15029 | 116 kDa U5 small nuclear ribonucleoprotein component | ○ | | | mRNA splicing |
| FUS | P35637 | RNA-binding protein FUS | ○ | | | mRNA splicing |
| HNRNPA3 | P51991 | Heterogeneous nuclear ribonucleoprotein A3 | ○ | | | mRNA splicing |
| HNRNPM | P52272 | Heterogeneous nuclear ribonucleoprotein M | | | ○ | mRNA splicing |
| HNRNPR | O43390 | Heterogeneous nuclear ribonucleoprotein R | | ○ | ○ | mRNA splicing |
| LSM2 | Q9Y333 | U6 snRNA-associated Sm-like protein LSm2 | ○ | | | mRNA splicing |
| LSM8 | O95777 | U6 snRNA-associated Sm-like protein LSm8 | ○ | | | mRNA splicing |
| PCBP1 | Q15365 | Poly(rC)-binding protein 1 | ○ | | | mRNA splicing |
| PCBP2 | Q15366 | Poly(rC)-binding protein 2 | | ○ | | mRNA splicing |
| PTBP1 | P26599 | Polypyrimidine tract-binding protein 1 | ○ | | ○ | mRNA splicing |
| PUF60 | Q9UHX1 | Poly(U)-binding-splicing factor PUF60 | | | ○ | mRNA splicing |
| RBM8A | Q9Y5S9 | RNA-binding protein 8A | | | ○ | mRNA splicing |
| SART1 | O43290 | U4/U6.U5 tri-snRNP-associated protein 1 | ○ | | ○ | mRNA splicing |
| SF3A3 | Q12874 | Splicing factor 3A subunit 3 | | | ○ | mRNA splicing |
| SF3B2 | Q13435 | Splicing factor 3B subunit 2 | | | ○ | mRNA splicing |
| SNRPE | P62304 | Small nuclear ribonucleoprotein E | ○ | | | mRNA splicing |
| SNW1 | Q13573 | SNW domain-containing protein 1 | ○ | | | mRNA splicing |
| SRRM2 | Q9UQ35 | Serine/arginine repetitive matrix protein 2 | ○ | | | mRNA splicing |
| SYMPK | Q92797 | Symplekin | ○ | | | mRNA splicing |
| U2SURP | O15042 | U2 snRNP-associated SURP motif-containing protein | | | ○ | mRNA splicing |
| YBX1 | P67809 | Y-box-binding protein 1 | ○ | ○ | | mRNA splicing |
| ACTR1A | P61163 | Alpha-centractin | | | ○ | Cell cycle |
| CDK1 | P06493 | Cyclin-dependent kinase 1 | ○ | ○ | ○ | Cell cycle |
| CDK4 | P11802 | Cyclin-dependent kinase 4 | ○ | | | Cell cycle |
| DCTN2 | Q13561 | Dynactin subunit 2 | | | ○ | Cell cycle |
| DYNC1LI2 | O43237 | Cytoplasmic dynein 1 light intermediate chain 2 | | ○ | ○ | Cell cycle |
| FEN1 | P39748 | Flap endonuclease 1 | | ○ | | Cell cycle |
| GOLGA2 | Q08379 | Golgin subfamily A member 2 | ○ | | ○ | Cell cycle |
| MAPK1 | Q16539 | Mitogen-activated protein kinase 14 | ○ | | | Cell cycle |
| MAPRE1 | Q15691 | Microtubule-associated protein RP/EB family member 1 | ○ | | | Cell cycle |
| MCM2 | P49736 | DNA replication licensing factor MCM2 | | | ○ | Cell cycle |
| MCM3 | P25205 | DNA replication licensing factor MCM3 | ○ | | | Cell cycle |
| MCM6 | Q14566 | DNA replication licensing factor MCM6 | ○ | | ○ | Cell cycle |
| MDC1 | Q14676 | Mediator of DNA damage checkpoint protein 1 | ○ | ○ | | Cell cycle |

**Table S2.** List of downregulated proteins associated with protein-protein interaction networks (PPI) in Figure 4 (continued).

| Gene name | Accession | Description | 4 h | 10 h | 16 h | PPI |
|---|---|---|---|---|---|---|
| MRE11A | P49959 | Double-strand break repair protein MRE11 | ○ | | | Cell cycle |
| NCAPH | Q15003 | Condensin complex subunit 2 | ○ | | ○ | Cell cycle |
| NEK7 | Q8TDX7 | Serine/threonine-protein kinase Nek7 | | ○ | ○ | Cell cycle |
| NIPBL | Q6KC79 | Nipped-B-like protein | | | ○ | Cell cycle |
| NPM1 | P06748 | Nucleophosmin | | | ○ | Cell cycle |
| NUMA1 | Q14980 | Nuclear mitotic apparatus protein 1 | | ○ | ○ | Cell cycle |
| NUP214 | P35658 | Nuclear pore complex protein Nup214 | | ○ | | Cell cycle |
| NUP93 | Q8N1F7 | Nuclear pore complex protein Nup93 | | ○ | | Cell cycle |
| ORC3 | Q9UBD5 | Origin recognition complex subunit 3 | | | ○ | Cell cycle |
| PDS5B | Q9NTI5 | Sister chromatid cohesion protein PDS5 homolog B | | | ○ | Cell cycle |
| POLE3 | Q9NRF9 | DNA polymerase epsilon subunit 3 | | ○ | | Cell cycle |
| PPP1R12A | O14974 | Protein phosphatase 1 regulatory subunit 12A | | | ○ | Cell cycle |
| PPP2R1A | P30153 | Serine/threonine-protein phosphatase 2A 65 kDa regulatory subunit A alpha isoform | | | ○ | Cell cycle |
| PSMA4 | P25789 | Proteasome subunit alpha type-4 | | | ○ | Cell cycle |
| PSMC1 | P62191 | 26S proteasome regulatory subunit 4 | ○ | | ○ | Cell cycle |
| PSMD1 | Q99460 | 26S proteasome non-ATPase regulatory subunit 1 | ○ | | | Cell cycle |
| PSMD14 | O00487 | 26S proteasome non-ATPase regulatory subunit 14 | | | ○ | Cell cycle |
| PSME3 | P61289 | Proteasome activator complex subunit 3 | ○ | | | Cell cycle |
| RANGAP1 | P46060 | Ran GTPase-activating protein 1 | | | ○ | Cell cycle |
| RCC2 | Q9P258 | Protein RCC2 | ○ | | | Cell cycle |
| RUVBL2 | Q9Y230 | RuvB-like 2 | | ○ | ○ | Cell cycle |
| SMC1A | Q14683 | Structural maintenance of chromosomes protein 1A | | | ○ | Cell cycle |
| SMC3 | Q9UQE7 | Structural maintenance of chromosomes protein 3 | | | ○ | Cell cycle |
| TP53BP1 | Q12888 | TP53-binding protein 1 | ○ | ○ | | Cell cycle |
| TUBB | P07437 | Tubulin beta chain | ○ | | | Cell cycle |
| UBE2N | P61088 | Ubiquitin-conjugating enzyme E2 N | ○ | | | Cell cycle |
| YWHAB | P31946 | 14-3-3 protein beta/alpha | ○ | | | Cell cycle |
| NUP107 | P57740 | Nuclear pore complex protein Nup107 | ○ | | | Cell cycle/ RNA transport |
| NUP188 | Q5SRE5 | Nucleoporin NUP188 homolog | ○ | | | Cell cycle/ RNA transport |
| NUP214 | P35658 | Nuclear pore complex protein Nup214 | | ○ | | Cell cycle/ RNA transport |
| TRNT1 | Q96Q11 | CCA tRNA nucleotidyltransferase 1 | ○ | | | RNA transport |
| EIF2S1 | P05198 | Eukaryotic translation initiation factor 2 subunit 1 | ○ | | | RNA transport |

**Table S2.** List of downregulated proteins associated with protein-protein interaction networks (PPI) in Figure 4 (continued).

| Gene name | Accession | Description | 4 h | 10 h | 16 h | PPI |
|---|---|---|---|---|---|---|
| PRMT5 | O14744 | Protein arginine N-methyltransferase 5 | ○ | | | RNA transport |

| | | | | |
|---|---|---|---|---|
| XPOT | O43592 | Exportin-T | ○ | RNA transport |
| EIF3C | Q99613 | Eukaryotic translation initiation factor 3 subunit C | ○ | RNA transport |
| EEF1A1 | P68104 | Elongation factor 1-alpha 1 | ○ | RNA transport |
| DDX20 | Q9UHI6 | Probable ATP-dependent RNA helicase DDX20 | ○ | RNA transport |
| NUPL1 | Q9BVL2 | Nucleoporin p58/p45 | ○ | RNA transport |
| SEH1L | Q96EE3 | Nucleoporin SEH1 | ○ | RNA transport |
| RPP38 | P78345 | Ribonuclease P protein subunit p38 | ○ | RNA transport |

**Table S3.** List of upregulated proteins associated with protein-protein interaction networks (PPI) in Figure 4.

| Gene name | Accession | Description | 4 h | 10 h | 16 h | PPI |
|---|---|---|---|---|---|---|
| AHCY | P23526 | Adenosylhomocysteinase | | | ○ | Immune system |
| ATP1B3 | P54709 | Sodium/potassium-transporting ATPase subunit beta-3 | | | ○ | Immune system |
| ATP6V0C | P27449 | V-type proton ATPase 16 kDa proteolipid subunit | ○ | | | Immune system |
| BST2 | Q10589 | Bone marrow stromal antigen 2 | | ○ | | Immune system |
| C4BPB | P20851 | C4b-binding protein beta chain | | | ○ | Immune system |
| CCT8 | P50990 | T-complex protein 1 subunit theta | ○ | | | Immune system |
| CD55 | P08174 | Complement decay-accelerating factor | ○ | ○ | ○ | Immune system |
| CD81 | P60033 | CD81 antigen | ○ | ○ | | Immune system |
| CD97 | P48960 | CD97 antigen | | | ○ | Immune system |
| CTSB | P07858 | Cathepsin B | ○ | | | Immune system |
| CTSL | P07711 | Cathepsin L1 | | ○ | ○ | Immune system |
| DDOST | P39656 | Dolichyl-diphosphooligosaccharide--protein glycosyltransferase 48 kDa subunit | ○ | | | Immune system |
| DEGS1 | O15121 | Sphingolipid delta(4)-desaturase DES1 | | | ○ | Immune system |
| DNAJC5 | Q9H3Z4 | DnaJ homolog subfamily C member 5 | | | ○ | Immune system |
| DNASE2 | O00115 | Deoxyribonuclease-2-alpha | | | ○ | Immune system |
| ECSIT | Q9BQ95 | Evolutionarily conserved signaling intermediate in Toll pathway mitochondrial | | ○ | | Immune system |
| H2BFS | P57053 | Histone H2B type F-S | | | ○ | Immune system |
| HIST1H2BC | P62807 | Histone H2B type 1-C/E/F/G/I | | | ○ | Immune system |
| HIST1H2BK | O60814 | Histone H2B type 1-K | | | ○ | Immune system |
| ICAM1 | P05362 | Intercellular adhesion molecule 1 | | | ○ | Immune system |
| ITGA1 | P56199 | Integrin alpha-1 | | | ○ | Immune system |
| KDELR1 | P24390 | ER lumen protein-retaining receptor 1 | | | ○ | Immune system |
| MGST1 | P10620 | Microsomal glutathione S-transferase 1 | | ○ | | Immune system |
| PRKCSH | P14314 | Glucosidase 2 subunit beta | ○ | | | Immune system |
| SDCBP | O00560 | Syntenin-1 | | ○ | ○ | Immune system |
| SLC3A2 | P08195 | 4F2 cell-surface antigen heavy chain | | | ○ | Immune system |
| SQSTM1 | Q13501 | Sequestosome-1 | | | ○ | Immune system |
| SURF4 | O15260 | Surfeit locus protein 4 | | | ○ | Immune system |
| TXNDC5 | Q8NBS9 | Thioredoxin domain-containing protein 5 | ○ | | | Immune system |
| H2BFS | P57053 | Histone H2B type F-S | | | ○ | Immune system/ DNA damage |
| HIST1H2BC | P62807 | Histone H2B type 1-C/E/F/G/I | | | ○ | Immune system/ DNA damage |
| HIST1H2BD | P58876 | Histone H2B type 1-D | ○ | ○ | ○ | DNA damage |
| HIST1H2BH | Q93079 | Histone H2B type 1-H | ○ | ○ | ○ | DNA damage |
| HIST1H2BM | Q99879 | Histone H2B type 1-M | ○ | ○ | ○ | DNA damage |
| HIST1H2BN | Q99877 | Histone H2B type 1-N | ○ | ○ | ○ | DNA damage |

**Table S3.** List of upregulated proteins associated with protein-protein interaction networks (PPI) in Figure 4 (continued).

| Gene name | Accession | Description | 4 h | 10 h | 16 h | PPI |
|---|---|---|---|---|---|---|

| | | | | |
|---|---|---|---|---|
| UQCRFS1 | P47985 | Cytochrome b-c1 complex subunit Rieske mitochondrial | ○ | Oxidative phosphorylation |
| MT-CO2 | P00403 | Cytochrome c oxidase subunit 2 | ○ | Oxidative phosphorylation |
| MT-ND4 | P03905 | NADH-ubiquinone oxidoreductase chain 4 | ○ | Oxidative phosphorylation |
| SDHC | Q99643 | Succinate dehydrogenase cytochrome b560 subunit mitochondrial | ○ | Oxidative phosphorylation |
